# Supplementary material for: Empowering integrative and collaborative exploration of single-cell and spatial multimodal data with SGS genome browser
Source: Cell Genom. 2025 Apr 14;5(5):100848. doi: 10.1016/j.xgen.2025.100848 (PMC12143324; doi:10.1016/j.xgen.2025.100848)
Supplement: Document S1. Figures S1–S13 and Table S1 [file mmc1.pdf]

Cell Genomics, Volume *5*

Supplemental information

Empowering integrative and collaborative exploration of single-cell and spatial multimodal data with SGS genome browser

Tingting Xia (夏婷婷), Jiahe Sun (孙家贺), Yongjiang Luo (罗永江), Hailong Guo (郭海龙), Yudi Mao (毛禹狄), Ling Xu (徐凌), Fang Lu (鲁方), and Yi Wang (王翊)

Supplementary Figures

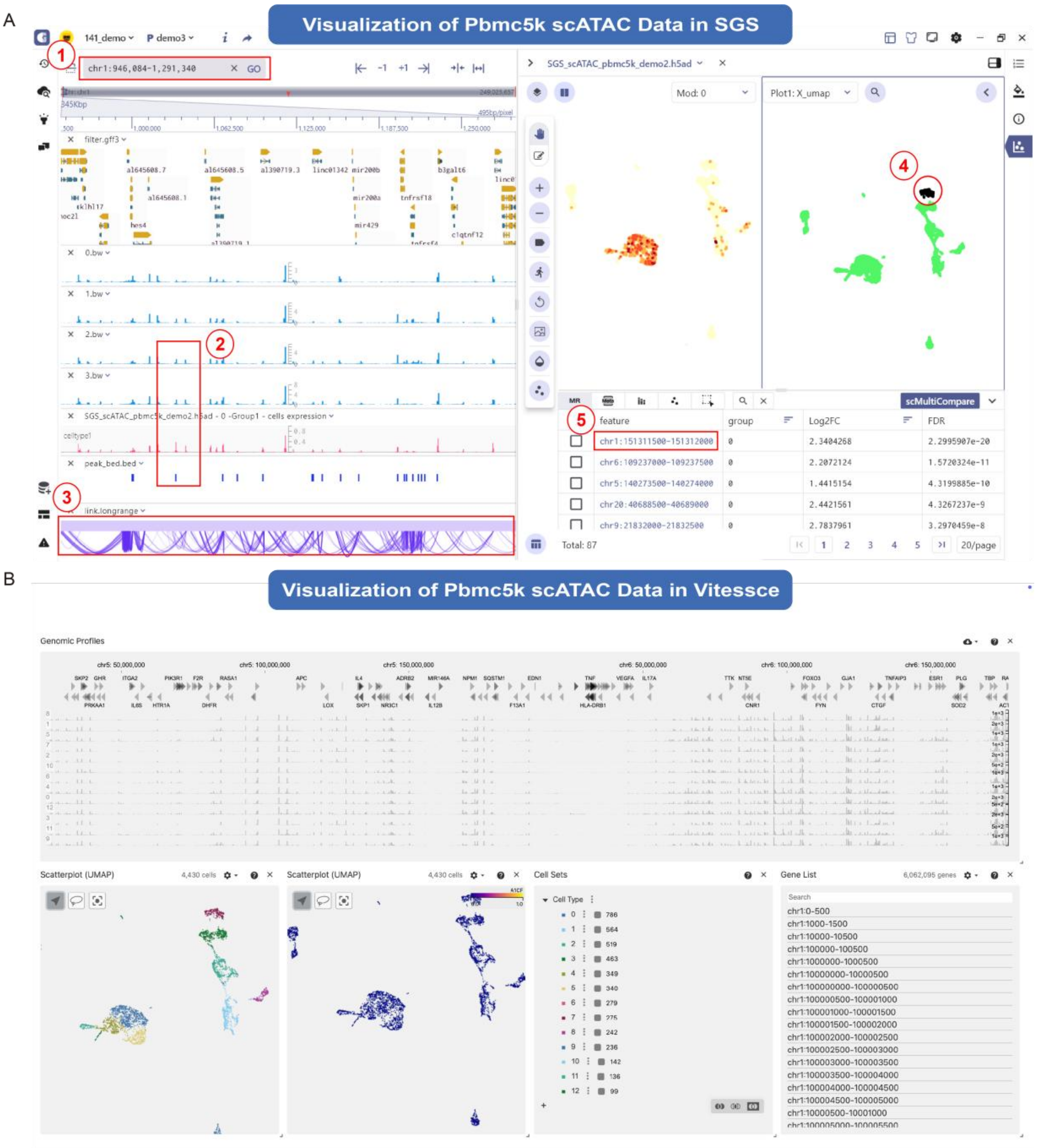

Figure S1. Comparative visualization of SGS and Vitessce for pbmc5k scATAC data, Related to Figure 1. (**A**) Screenshot of SGS visualization for pbmc5k scATAC data (demo link). Compared to Vitessce, SGS excels in gene-CRE links visualization, multi-panel coordination, and rapid navigation functions. SGS enables interactive visualization of pbmc5k scATAC data, allowing users to click on peaks in the single-cell panel to explore chromatin accessibility heterogeneity across cell types and navigate to associated genomic regions (**marked as 1 and 5**). Users can select specific cell clusters in the single-cell panel, and the genome browser will immediately generate the corresponding epigenomic information (**marked as 2 and 4**). SGS also supports the visualization of gene-CRE (gene-cis-regulatory element) links, highlighting interaction strengths through varying opacity curves (**marked as 3**). In summary, SGS's superiority in gene-CRE links visualization, multi-panel coordination, and rapid navigation functions. (**B**) Screenshot of Vitessce visualization for pbmc5k scATAC data (demo link). Vitessce provides basic visualization of single-cell embeddings and genome-mapped signals (gene structure and chromatin accessibility). However, it has

limitations in multi-panel coordination between single-cell and genome browser, cannot link peak cell expression heterogeneity to epigenome tracks, and does not support dynamic updates of chromatin accessibility genome-mapped signals based on cell cluster selection in single-cell view. Additionally, Vitessce lacks the ability to visualize gene-CRE links.

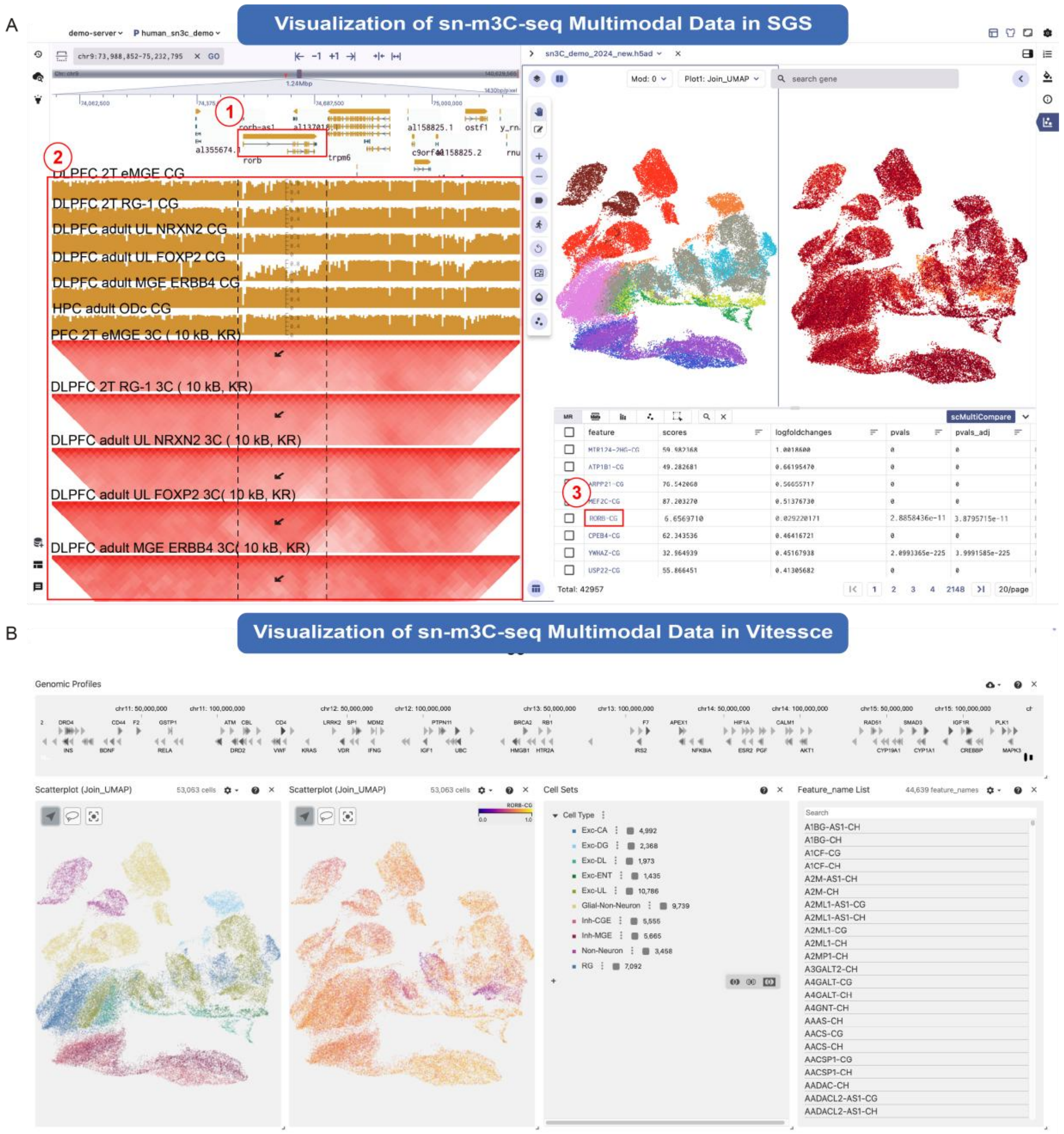

Figure S2. Comparison visualization of sn-m3C-seq data between SGS and Vitessce, Related to Figure 4A. (**A**) Human sn-m3C-seq data visualization of SGS (demo link). SGS can integrate complex sn-m3C-seq epigenomic multimodal data into a unified interface for visualization, offering significant functional advantages over Vitessce, as detailed below. SGS supports the display of cell clustering alongside genome-mapped tracks such as CG methylation signals and HiC tracks for specific cell types (**marked as 2**). SGS features a multi-panel coordination design and adaptive communication mechanism that enhances the sn-m3C-seq data exploration. Users can click on specific features in the single-cell marker table (such as the *RORB* gene) to quickly navigate to the corresponding genome region and obtain multi-level information, including chromatin accessibility, HiC interaction intensity, and methylation signal distribution (**marked as 1 and 3**). Additionally, SGS supports multiple HiC normalization methods (e.g., KR, VC, VC_SQRT) and custom interaction intensity thresholds for data filtering. (**B**) The visualization of sn-m3C-seq multimodal data in Vitessce (demo link). Vitessce currently does not support the integrated visualization of sn-m3C-seq data within a

unified interface. Vitessce lacks co-visualization of these epigenomic genome-mapped signals with single-cell atlas, and cannot rapidly localize marked features. Vitessce's components currently cannot offer HiC and methylation data normalization and filtering functions.

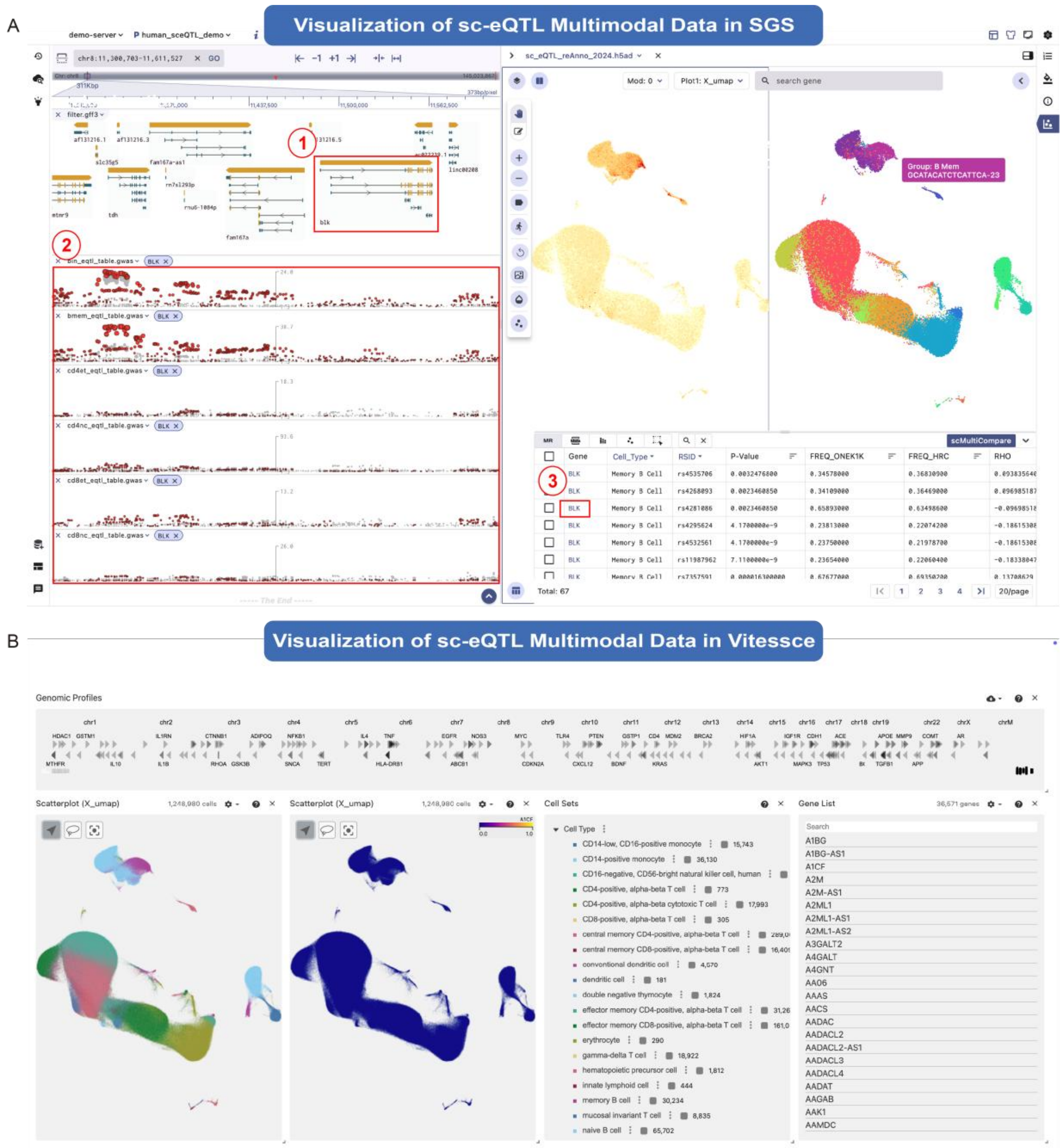

Figure S3. Comparison visualization of sc-eQTL data between SGS and Vitessce, Related to Figure 5A. (**A**) The screenshot of OneK1K sc-eQTL visualization demo of SGS (demo link). SGS's advantages in displaying associations between gene expression and genetic variation and in data filtering and navigation make it a better choice than Vitessce in visualizing sc-eQTL multimodal data. SGS integrates single-cell panels with the genome browser using sc-eGenes (genes significantly associated with sc-eQTL) as anchors. This allows for efficient identification of sc-eQTL regions linked to cell type-specific expression (**marked as 2 and 3**). For instance, clicking on a gene (e.g., *BLK*) in the single-cell panel reveals its heterogeneous expression across cell types and navigates to the corresponding sc-eQTL region, highlighting significant loci in red in the genome browser. SGS also supports robust genome navigation and query functions. Users can locate specific sc-eQTL regions by entering gene symbols (e.g., *BLK*) or genomic positions (e.g., chr8:11300703-11611527) and view detailed information such as p-values and site IDs. Additionally, users can set p-value thresholds to filter sc-eQTL regions (**marked as 1**). (**B**) The visualization of sc-eQTL

multimodal data in Vitessce (demo link). Vitessce supports the visualization of the sc-eQTL data cell atlas. However, Vitessce currently lacks the ability to display sc-eQTL regions and offers limited integration, requiring manual zooming, dragging, and filtering, which hinders user experience.

A

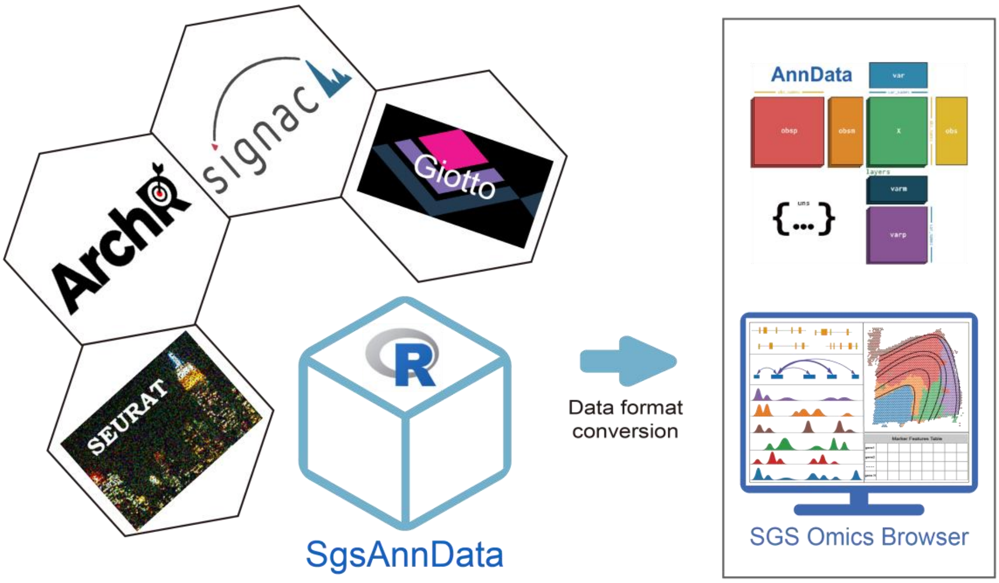

B

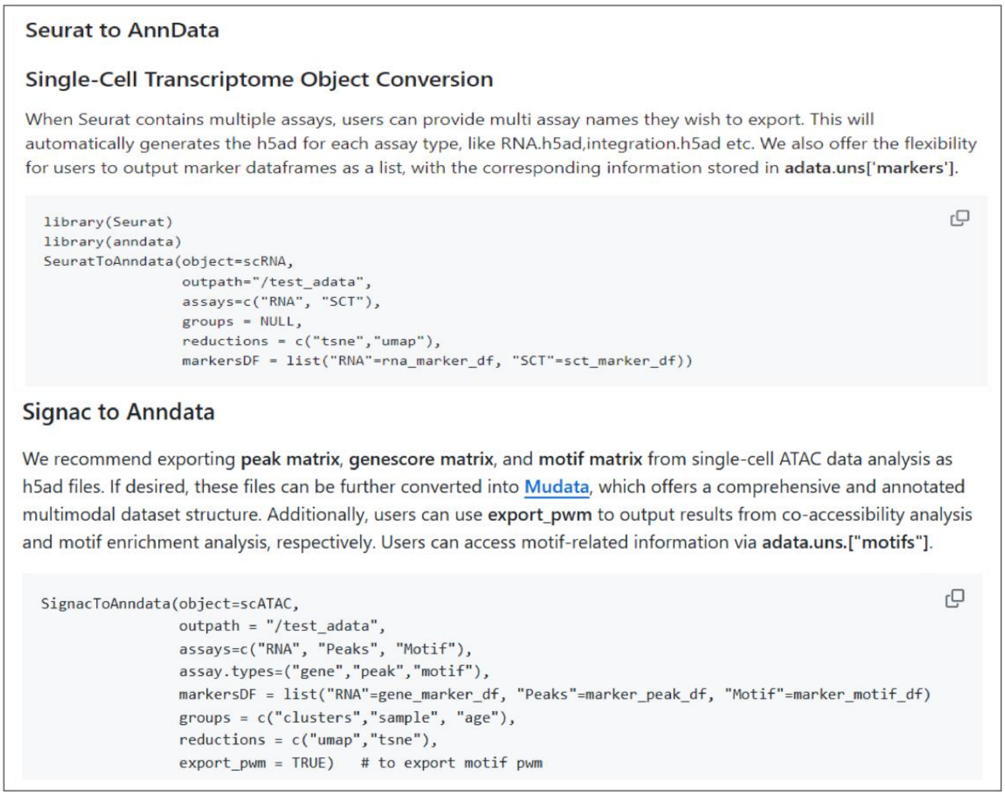
Seurat to AnnData

Single-Cell Transcriptome Object Conversion

When Seurat contains multiple assays, users can provide multi assay names they wish to export. This will automatically generates the h5ad for each assay type, like RNA.h5ad,integration.h5ad etc. We also offer the flexibility for users to output marker dataframes as a list, with the corresponding information stored in **adata.uns['markers']**.

```
library(Seurat)
library(anndata)
SeuratToAnndata(object=scRNA,
                outpath="/test_adata",
                assays=c("RNA", "SCT"),
                groups = NULL,
                reductions = c("tsne","umap"),
                markersDF = list("RNA"=rna_marker_df, "SCT"=sct_marker_df))
```

Signac to Anndata

We recommend exporting **peak matrix**, **genescore matrix**, and **motif matrix** from single-cell ATAC data analysis as h5ad files. If desired, these files can be further converted into Mudata, which offers a comprehensive and annotated multimodal dataset structure. Additionally, users can use **export_pwm** to output results from co-accessibility analysis and motif enrichment analysis, respectively. Users can access motif-related information via **adata.uns.["motifs"]**.

```
SignacToAnndata(object=scATAC,
                outpath = "/test_adata",
                assays=c("RNA", "Peaks", "Motif"),
                assay.types=("gene","peak","motif"),
                markersDF = list("RNA"=gene_marker_df, "Peaks"=marker_peak_df, "Motif"=marker_motif_df)
                groups = c("clusters","sample", "age"),
                reductions = c("umap","tsne"),
                export_pwm = TRUE)   # to export motif pwm
```

Figure S4. SgsAnnData R package of SGS, Related to Figure 1. (**A**) SgsAnnData of SGS used for the conversion of single-cell analysis objects from tools like Seurat, Giotto, Signac, and ArchR into the AnnData format. (**B**) The SeuratToAnndata function converts Seurat objects into h5ad files, with parameters including assay, metadata, reduction, outpath, etc. The SignacToAnndata function is used to convert Signac objects into multiple h5ad files, which are then stored in MuData format. To perform this conversion, users need to utilize the SignacToAnndata function and provide parameters such as assay, metadata, reduction, motifs, markers etc.

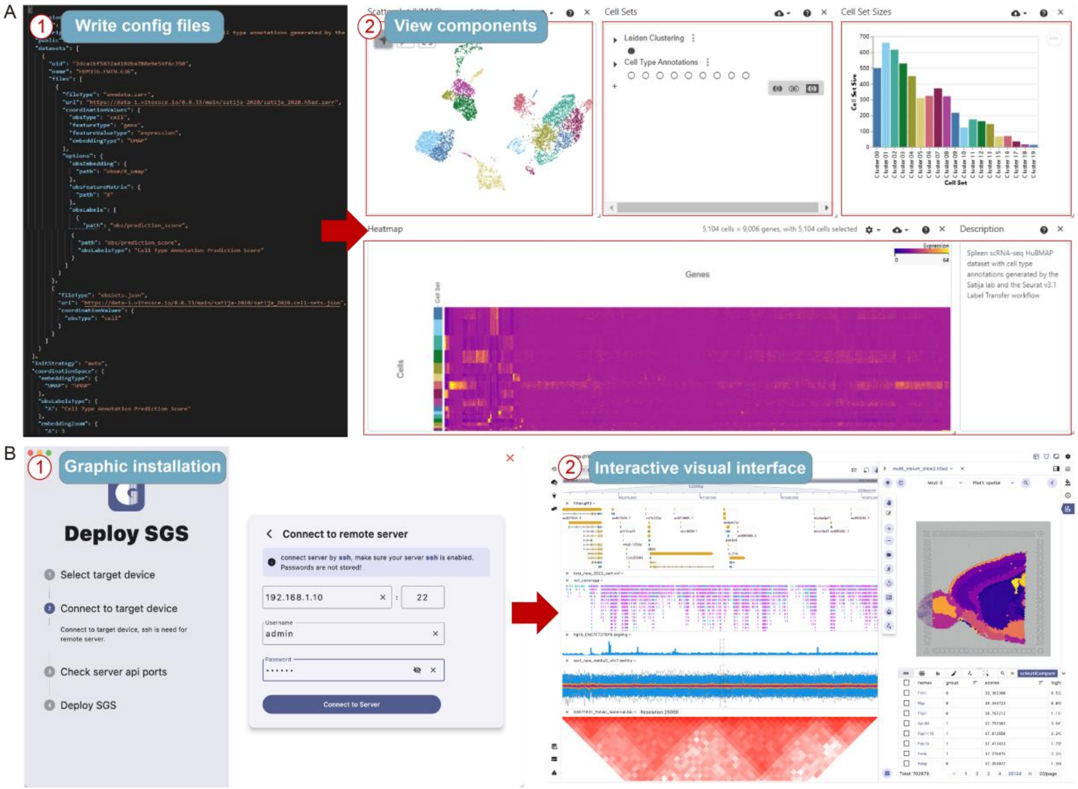

Figure S5. Comparative analysis of installation and interface usability between SGS and Vitessce, Related to STAR Methods. (**A**) Vitessce involves a more complex configuration process and requires detailed interface settings, reflecting a more technical approach to setup and usability. (**B**) SGS features a streamlined graphical installation process and user-friendly interface operations, designed to enhance ease of use and accessibility.

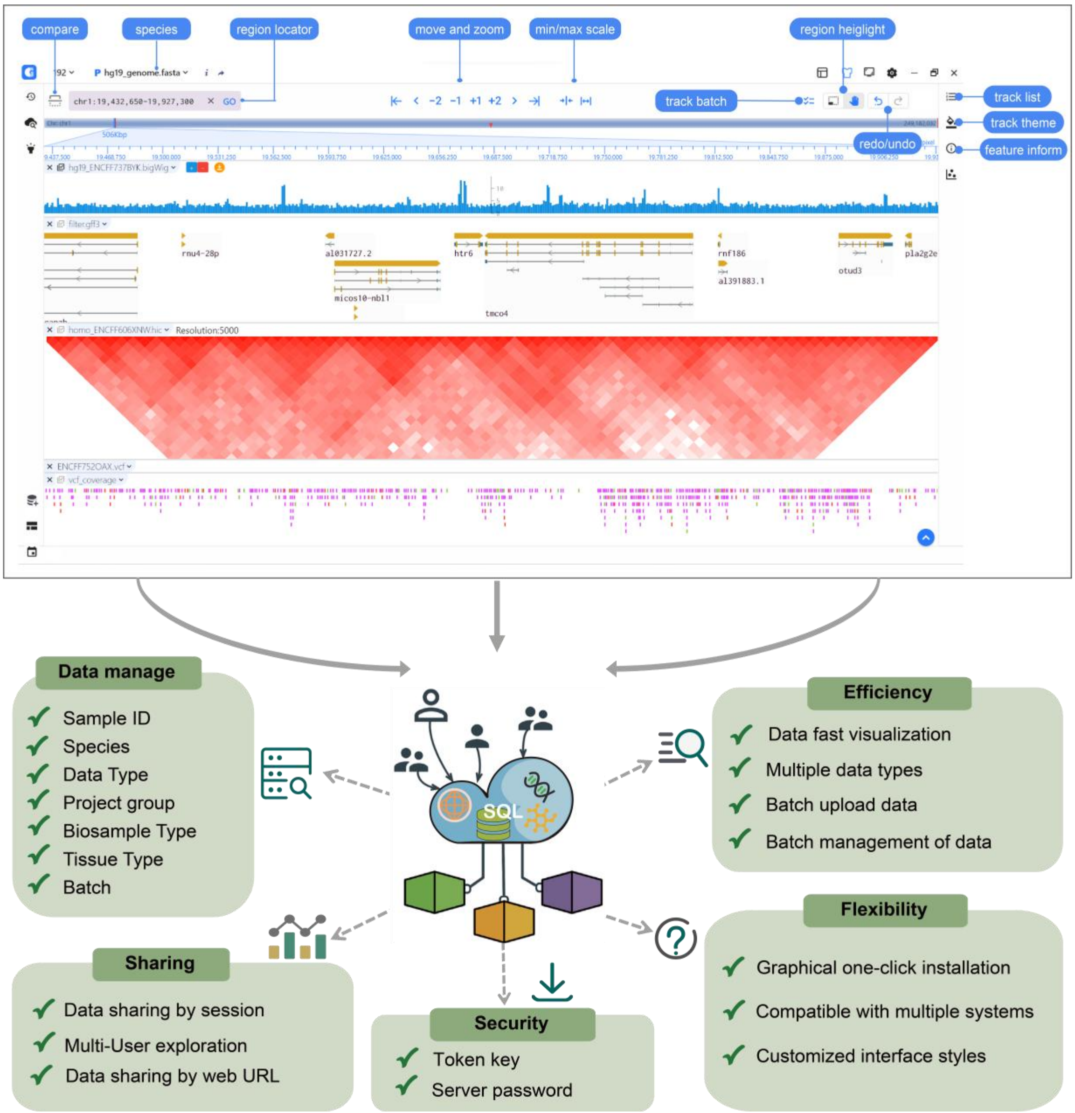

Figure S6. Graphical installation and interface operability of SGS, Related to STAR Methods. SGS features a highly user-friendly graphical installation process. Additionally, it excels in multiple aspects: seamless data management, efficient data sharing, robust security and privacy protection, high-performance visualization efficiency, and remarkable software flexibility.

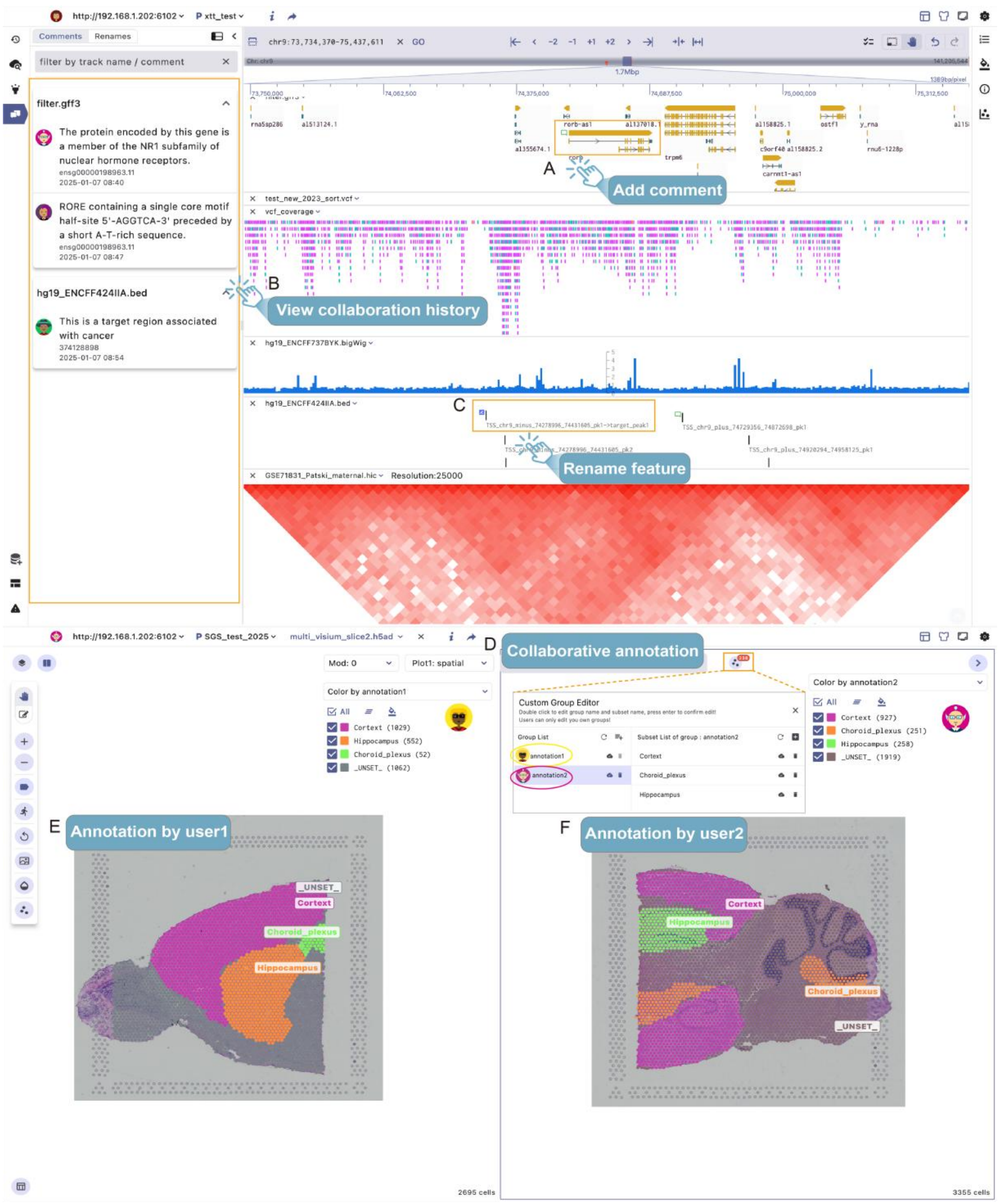

Figure S7. Collaborative visualization features of SGS, Related to STAR Methods. (**A**) Genome-mapped data collaboration. SGS supports multiple users in adding comments to features. (**B**) Viewing collaboration history. SGS allows users to track changes and interactions in the collaborative visualization process. (**C**) Renaming features. SGS enables users to rename features for collaborative visualization. (**D**) Collaborative cell annotation. SGS allows multiple users to create annotation groups. (**E,F**) Comparing annotation results. SGS enables users to compare annotations from different users (e.g., User 1 vs. User 2).

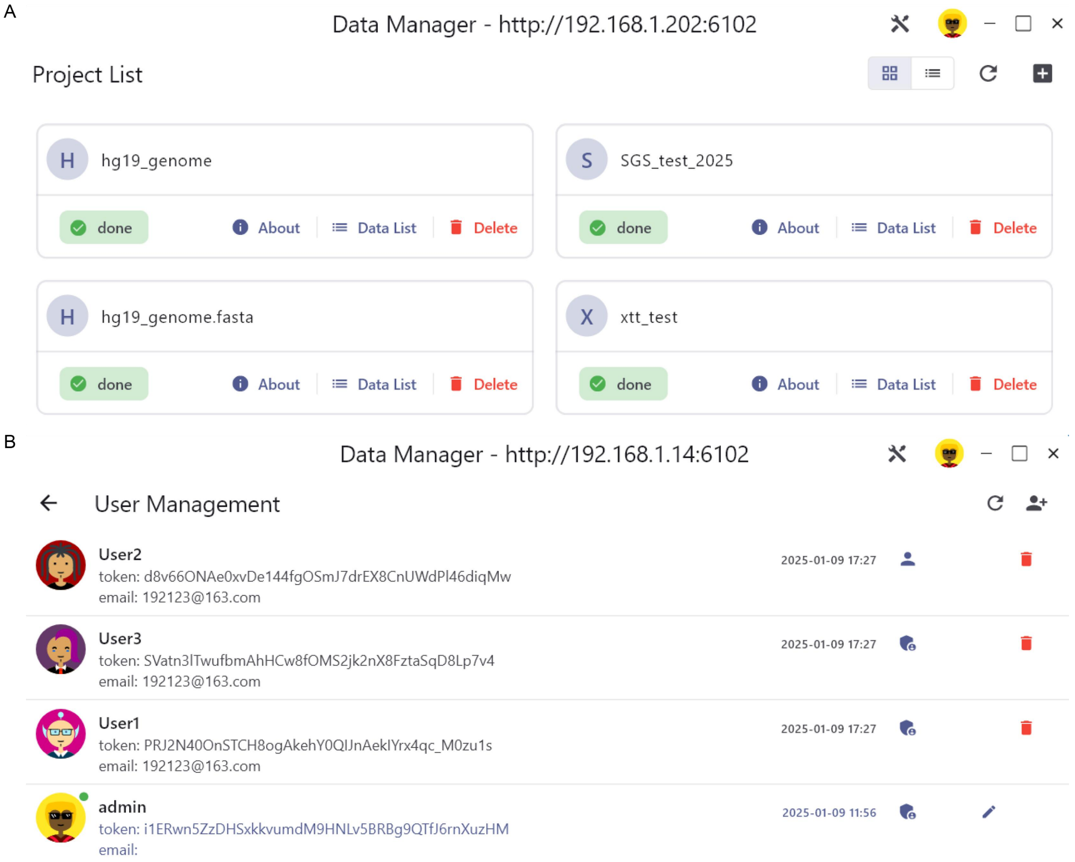

Figure S8. Screenshot of SGS data and user management interface, Related to STAR Methods. (**A**) Screenshot of the data management interface. (**B**) Screenshot of the user management interface.

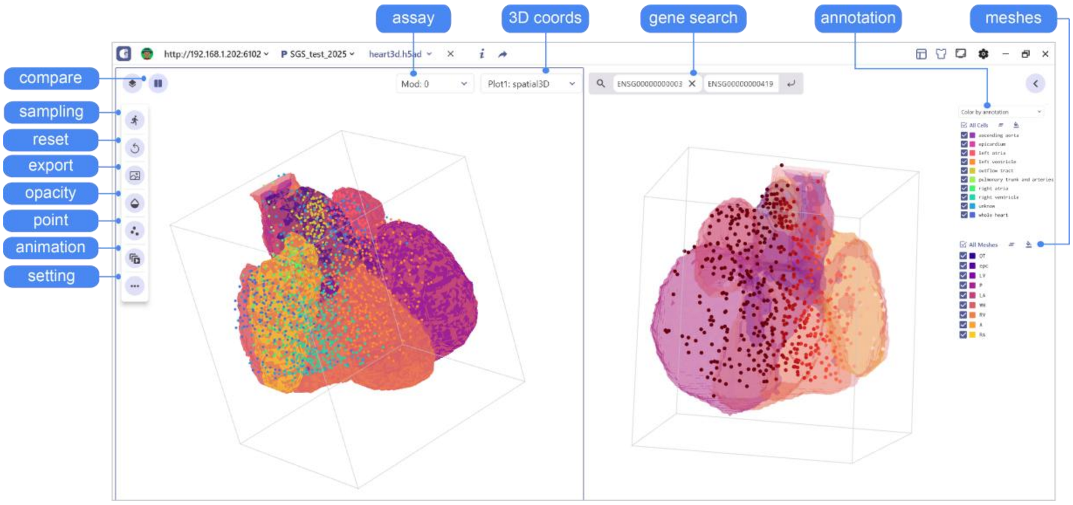

Figure S9. The visualization panel of 3D SRT data, Related to Figure 2.

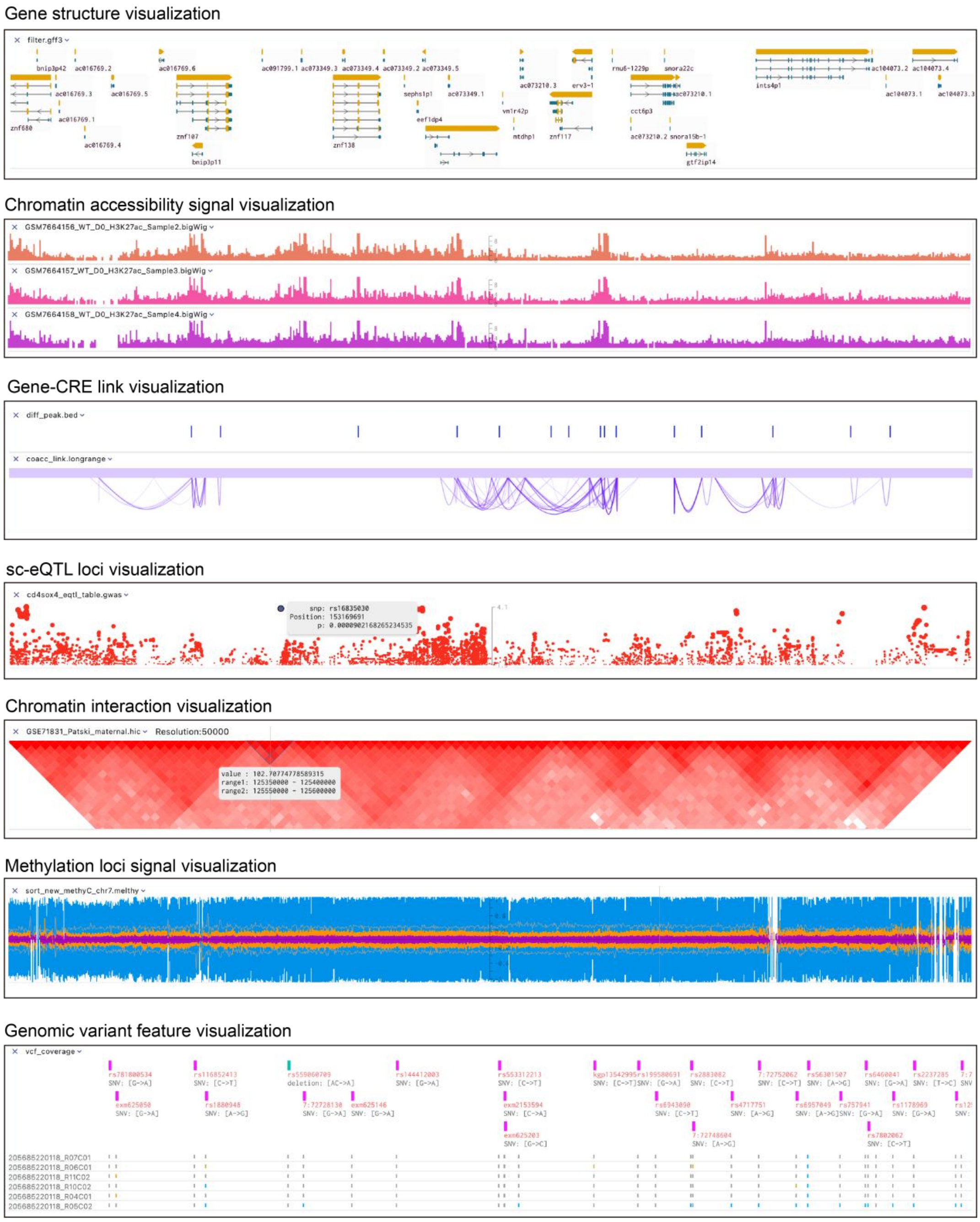

Figure S10. Screenshot of genome tracks in the SGS genome browser framework, Related to Figure 3. Gene structure, chromatin accessibility signal, gene-CRE link, sc-eQTL loci, chromatin interaction, methylation loci signal, and genomic variant feature are displayed from top to bottom.

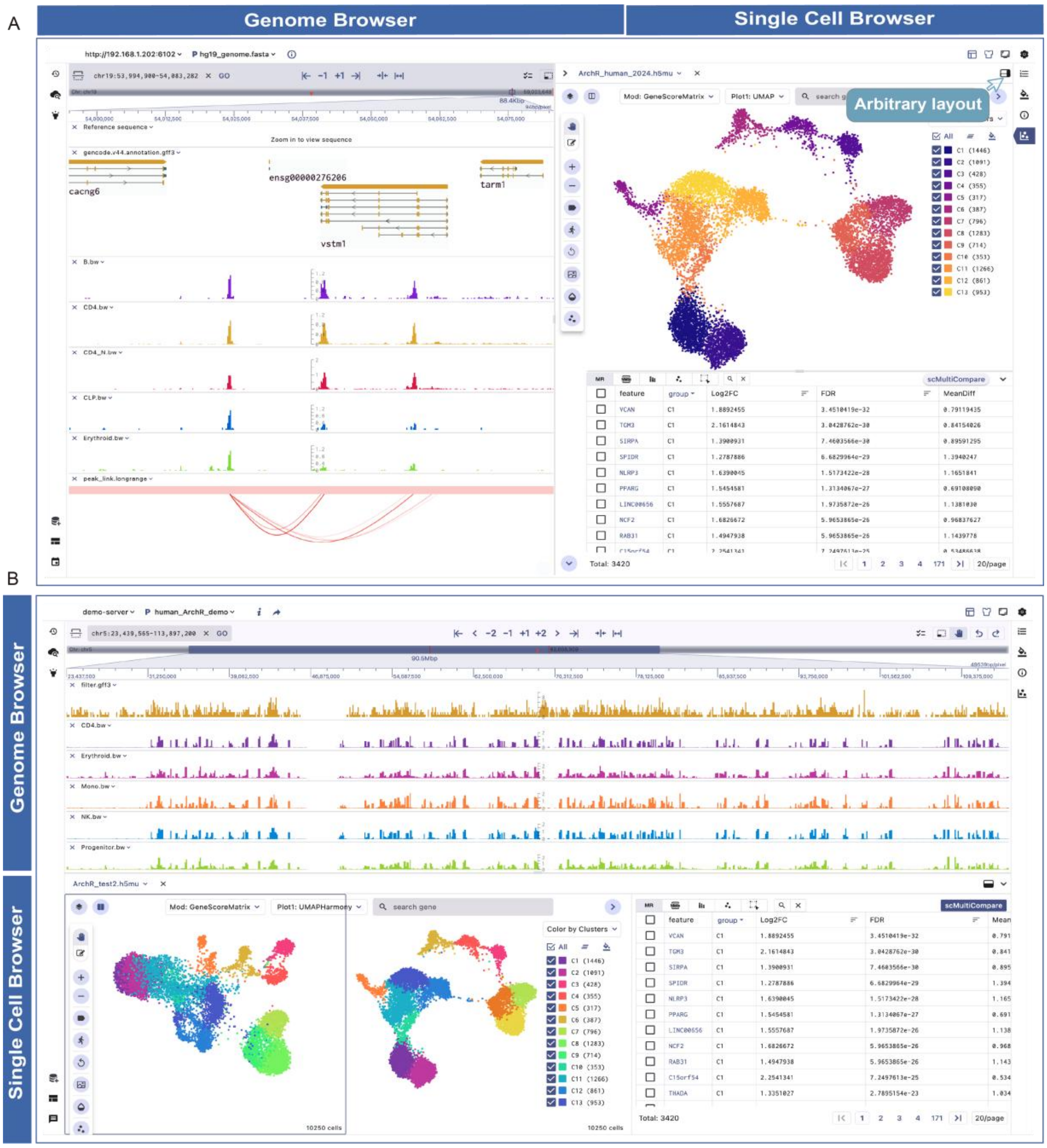

Figure S11. Flexible interface layout of SG visualization module, Related to Figure 3. The depicted figure showcases the SG_H and SG_V layout modes of the SG module. (**A**) The SG_H layout mode emphasizes assisting users in comparing multiple tracks in specific genomic regions, such as chromosome gene annotations and epigenetic modification signals, with single-cell omics information. (**B**) The SG_V layout mode vertically arranges the single-cell data and genome information, allowing users to obtain a view with fewer genome track details across the entire chromosome.

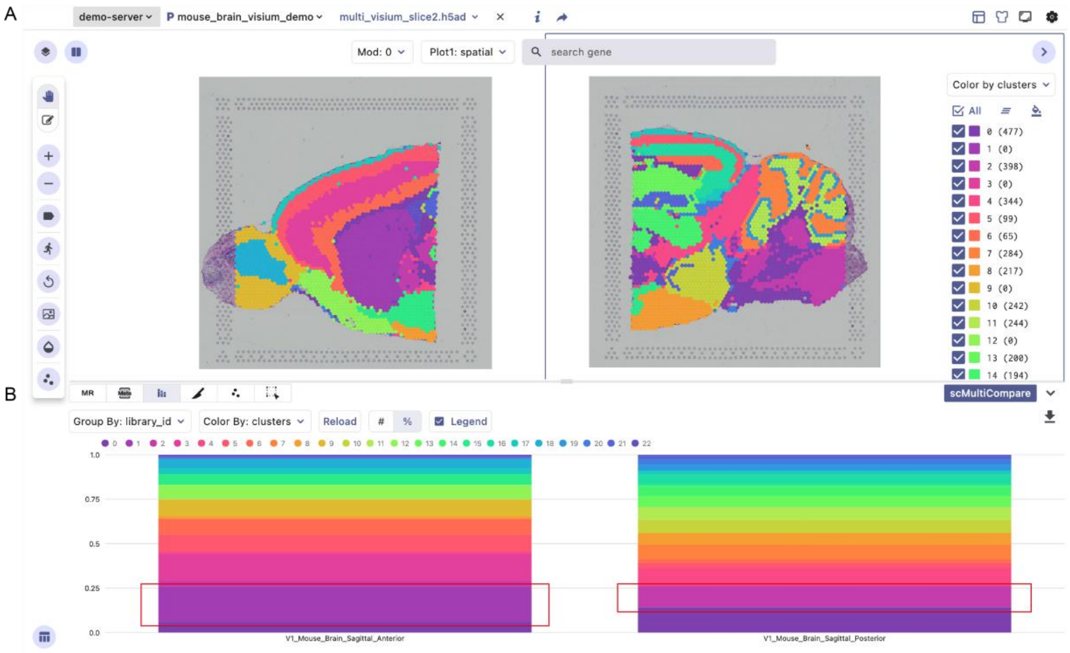

Figure S12. The SC module displays 10x Visium data of the anterior and posterior mouse brain, Related to Figure 4B. (**A**) The left and right panels present the cell clusters of the anterior and posterior brain spatial slices respectively. (**B**) The cell proportion plots were generated for the mouse brain, grouped by sample ID.

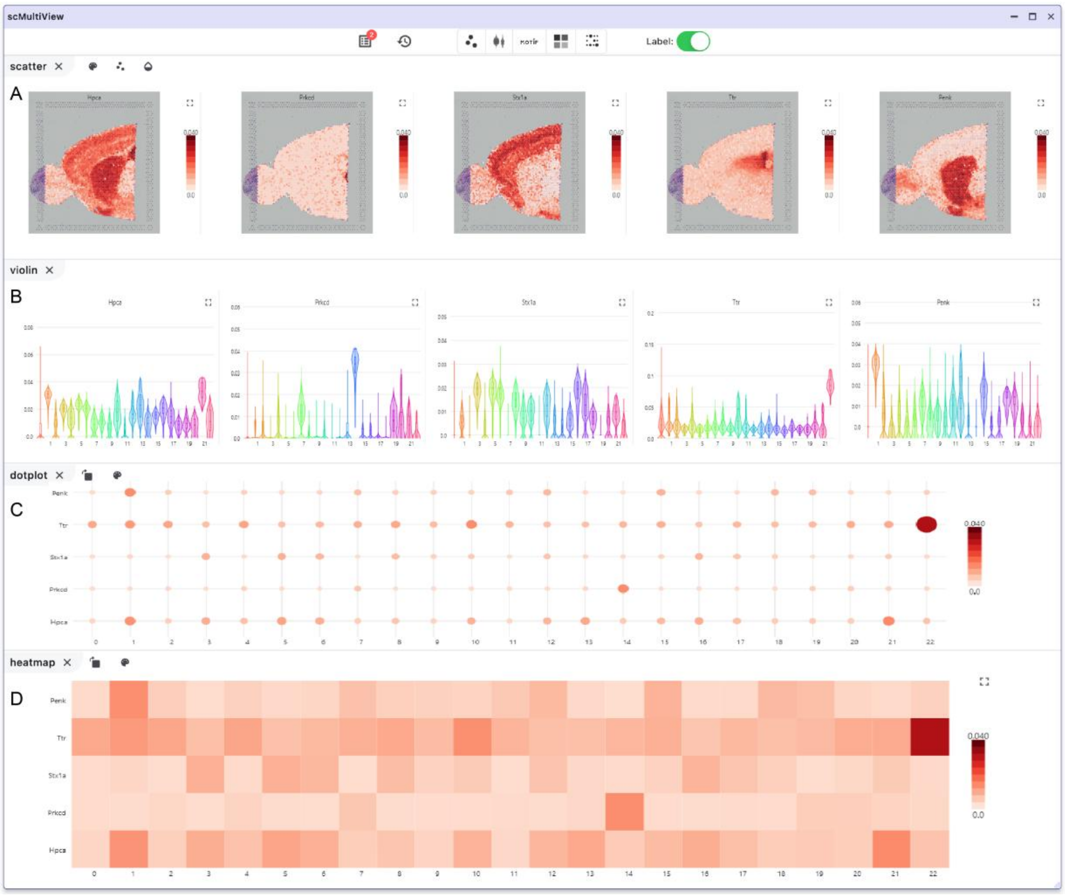

Figure S13. Visualizing development-related genes (*Hpca*, *Prkcd*, *Stx1a*, *Ttr*, *Penk*) in 10x Visium mouse brain data using scMultiView, Related to Figure 4B. (**A**) Spatial feature plot of the selected gene. (**B**) Violin plot displaying gene expression levels across clusters. (**C**) Dot plot of the selected gene. (**D**) Heatmap visualizing gene expression across different cell clusters.

Supplementary Tables

Table S1. Comparison of SGS with existing mainstream scMulti-omics data visualization tools, Related to Figure 1.

	Comparison item	SGS	Vitessce	AtlasXplore	Cellxgene	Loom-Viewer	Single Cell Explorer	UCSC Cell Browser	Loupe Browser	ST Viewer	TissUUmaps3
Visualization Function	Spatial feature visualization	✓	✓	✓	✓	✓			✓	✓	✓
	Highlight gene expression	✓	✓	✓	✓	✓	✓	✓	✓	✓	✓
	Highlight metadata	✓	✓	✓	✓	✓	✓	✓	✓	✓	✓
	Tissue image visualization	✓	✓		✓				✓	✓	✓
	Multi-feature comparison	✓	✓	✓	✓	✓		✓	✓		
	Multi-slice visulization	✓	✓		✓				✓		✓
	Multiple embeddings	✓	✓	✓	✓	✓	✓	✓	✓	✓	✓
	Gene structure visulization	✓	✓	✓							
	Chromatin accessibility signal	✓	✓	✓							
	Meta table	✓	✓	✓	✓						
	3D transcriptomic data visualization	✓									
	Feature sequence visualization	✓									
	Track information	✓									
	sc-eQTL loci visualization	✓									
	Methylation loci Visualization	✓									
	Gene-CRE link visulization	✓									
	Motif logo visulization	✓									
	Variant feature visualization	✓									
	Chromatin interaction visualization	✓									
	Dual-chromosome comparative visualization	✓									
Interactivity	Cell selection and export	✓	✓	✓	✓		✓	✓	✓	✓	✓
	Move/Zoom	✓	✓	✓	✓		✓	✓	✓	✓	✓
	Image setting	✓	✓		✓				✓	✓	✓
	Global theme setting	✓									
	Track Feature search (gene/snp/region)	✓		✓							
	Track scale/statistics	✓									
	Graphical installation	✓							✓	✓	
Views coordinations	Single-cell views coordinations	✓	✓	✓				✓	✓		
	Single-cell view and genome browser coordinations	✓		✓							
User Collaboration	Web Sharing	✓	✓	✓	✓	✓	✓	✓			✓
	Session URL sharing	✓			✓			✓			
	Create and manage projects	✓									
	Add and delete data in batches	✓									
	Data organization and classification	✓									
	Track comments	✓									
	Real-time annotation	✓					✓				
	Authority management	✓									
Data Format	anndata/anndata.zarr	✓	✓		✓		✓				✓
	mudata/mudata.zarr	✓	✓								
	GFF	✓									
	VCF	✓									
	BED	✓	✓	✓							
	BigWig	✓	✓	✓							
	MethylC	✓									
	Longrange	✓									
	BigInteract	✓									
	HiC	✓									
	GWAS	✓									
